# Transversus Abdominis Plane Block Reduces Intraoperative Opioid Consumption in Patients Undergoing Periacetabular Osteotomy

**DOI:** 10.3390/jcm11174961

**Published:** 2022-08-24

**Authors:** Jannis Löchel, Georgi I. Wassilew, Michael Krämer, Christopher Kohler, Robert Karl Zahn, Vincent Justus Leopold

**Affiliations:** 1 Charité–Universitätsmedizin Berlin, Corporate Member of Freie Universität Berlin, Humboldt-Universität zu Berlin, and Berlin Institute of Health, Center for Musculoskeletal Surgery Augustenburger Platz 1, D–13353 Berlin, Germany; 2 Department for Orthopaedic Surgery, University of Greifswald, Ferdinand-Sauerbruch-Straße, D–17475 Greifswald, Germany; 3 Charité–Universitätsmedizin Berlin, Corporate Member of Freie Universität Berlin, Humboldt-Universität zu Berlin, and Berlin Institute of Health, Clinic for Anesthesiology and Intensive Care, Charitéplatz 1, D–10117 Berlin, Germany; 4 Orthopädisches Versorgungszentrum Zehlendorf, Clayallee 225A, D–14195 Berlin, Germany

**Keywords:** periacetabular osteotomy, pain management, TAP block, intraoperative analgesia

## Abstract

Background: Administering intraoperative analgesia in patients undergoing periacetabular osteotomy (PAO) is challenging due to both the relevant surgical approach and osteotomies, which are associated with pain. The aim of this study was to assess the effect of the transversus abdominis plane block (TAPb) on intraoperative opioid consumption and circulation parameters in PAO patients. Patients and Methods: We conducted a two-group randomized-controlled trial involving 42 consecutive patients undergoing PAO for symptomatic developmental dysplasia of the hip (DDH) in our department. Patients assigned to the study group received an ultrasound-guided TAPb with 0.75% ropivacaine before the beginning of the surgery and after general anesthesia induction. Patients assigned to the control group did not receive a TAPb. General anesthesia was conducted according to a defined study protocol. The primary endpoint of the study was the intraoperative opioid consumption, measured in morphine equivalent dose (MED). Secondary endpoints were the assessment of intraoperative heart rate, mean arterial pressure (MAP), need for hypotension treatment, and length of hospital stay (LOHS). A total of 41 patients (*n* = 21 TAPb group, *n* = 20 control group) completed the study; of these, 33 were women (88.5%) and 8 were men (19.5%). The mean age at the time of surgery was 28 years (18–43, SD ± 7.4). All operations were performed by a single high-volume surgeon and all TAPb procedures were performed by a single experienced senior anesthesiologist. Results: We observed a significantly lower intraoperative opioid consumption in the TAPb group compared to the control group (930 vs. 1186 MED per kg bodyweight; *p* = 0.016). No significant differences were observed in the secondary outcome parameters. We observed no perioperative complications. Conclusion: Ultrasound-guided TAPb significantly reduces intraoperative opioid consumption in patients undergoing PAO.

## 1. Introduction

Locoregional analgesia techniques are routinely used to improve perioperative pain management. The transversus abdominis plane block (TAPb) infiltrates the Nn. ilioinguinalis, iliohypogastricus, and spinal nerves in the plane between the internal oblique and transversus abdominis muscles (IOAM and TAM, respectively) using local anesthetics (LA), providing anesthesia of the anterolateral abdominal wall [1]. TAPb is a long-established locoregional procedure for anesthesia in abdominal and retroperitoneal surgery, and more recently, it has been used in orthopedic surgery [2,3,4,5,6].

Periacetabular osteotomy (PAO) is the surgical treatment of choice for symptomatic developmental dysplasia of the hip (DDH) in young adults [7,8,9,10]. Over time, the exposure has been reduced through modifications to the surgical approach, however it remains a procedure with relevant surgical trauma causing significant intraoperative pain, mostly in opioid-naive young adults [11,12,13,14].

Selecting an effective intraoperative pain management regime with a low dosage of opioids to reduce the associated adverse effects and maintain stable circulation parameters remains challenging in patients undergoing PAO.

Besides general anesthesia, epidural anesthesia (EA) and psoas-compartment catheters are in use to provide intraoperative analgesia [15,16,17]. EA can lead to postoperative nausea and vomiting (PONV) and drowsiness [15,18]. Postoperative neurological examination and mobilization can also be impeded by EA. Furthermore, it can cause neuraxial complications [2,16]. Even though there are safe locoregional analgesia techniques for PAO patients, studies investigating the effectiveness of TAPb are lacking in the literature.

The aim of the study was to investigate the intraoperative effect of the TAPb. The primary endpoint of the study was intraoperative opioid consumption, measured in morphine equivalent dose (MED). Secondary endpoints were the assessment of intraoperative heart rate, mean arterial pressure (MAP), need for hypotension treatment, and length of hospital stay (LOHS). We hypothesized that the TAPb would reduce intraoperative opioid consumption. We further hypothesized that the intraoperative stability of MAP and heart rate would be at least equivalent to the control group.

## 2. Patients and Methods

**Figure 1:**
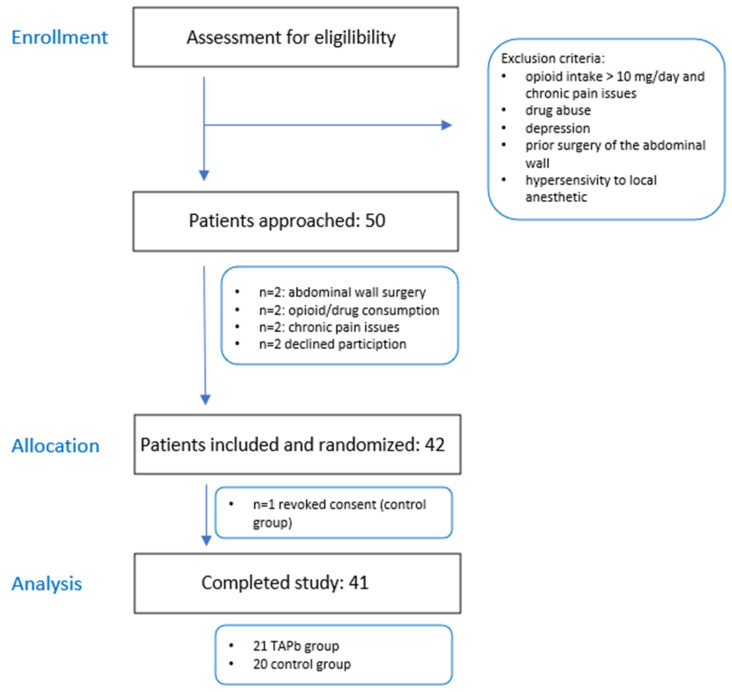
Flow chart.

Institutional review board approval and study registration were obtained for this two-group randomized-controlled exploratory trial (EA2/098/17) and Supplementary Materials (the CONSORT 2010 Checklist) completed. Fifty consecutive patients undergoing PAO for symptomatic DDH were approached for study participation during their preoperative consultation in the outpatient department. The exclusion criteria were: age < 18 years, inability to give informed consent, abdominal wall surgery in the case history, drug or analgesic misuse before the surgery (e.g., daily opioid intake ≥ 10 mg MED), depression, and documented hypersensitivity to LA. Of those excluded, two patients had undergone prior surgery of the abdominal wall, two patients were excluded due to preoperative opioid or drug consumption, two presented with chronic preoperative pain issues, and two declined study participation. Twenty-one patients were assigned to the TAPb group and 21 to the control group. One patient in the control group was later excluded from the study due to consent revocation (see Figure 1). Forty-one patients completed the study protocol.

Thirty-three patients were women (88.5%) and eight were men (19.5%). At the time of the surgery, the mean patient age was 28 years (18 to 43, SD 8) in the TAPb group and 28.5 years (18 to 39, SD 7) in the control group (*p* = 0.89). The mean body mass index (BMI) was 23 kg/m^2^ (15 to 31, SD 4) in the TAPb group and 24.5 kg/m^2^ (18 to 34, SD 3.6) in the control group (*p* = 0.34). A single high-volume surgeon (GIW) performed all the operations, and all TAPb procedures were performed by a single experienced senior anesthesiologist (MK). The overall opioid consumption was calculated according to the intraoperatively administered medication [19]. The computed randomization of the patients was kept in sealed envelopes prior to the clinical initiation of the study, and only revealed to the senior anesthesiologist after induction of the anesthesia before performing the TAPb. Unblinding of the patients was achieved 48 h after the surgery.

## 3. Anesthesia Protocol

The patients in both groups received general anesthesia with orotracheal intubation under a standardized protocol. Anesthesia induction was achieved using propofol (1–2.5 mg/kg) (B. Braun, Melsungen, Germany), fentanyl (0.5–1 µg/kg) (Hameln Pharma, Hameln, Germany), and cisartracurium (0.1–0.2 mg/kg) (GlaxoSmithKline, Brentford, UK). The group allocation was then revealed to the senior anesthesiologist performing the TAPb procedures for the patients of the study group. Intraoperative anesthesia maintenance was achieved using propofol (5–10 mg/kg/h) and fentanyl. In cases of intraoperative hypotension, the anesthetic depth was adjusted and 0.9% saline infusion (B. Braun, Melsungen, Germany) was administered. Whenever necessary, this was complemented by cafedrine/theodrenaline (Ratiopharm, Ulm, Germany). Hypertension was treated with an adjustment of the anesthetic depth and by administration of urapidil (Takeda Pharmaceutical, Tokio, Japan) when necessary. All patients were given tranexamic acid (1 g) (Pfizer, NY, USA) as a continuous infusion. Just before the end of surgery, all patients were given 0.1 mg/kg piritramide (Hameln Pharma, Hameln, Germany).

## 4. TAP Block Protocol

The patients in the TAPb group received an ultrasound-guided ipsilateral TAPb via a posterior approach. The TAPb procedure was performed in a supine position while visualizing the IOAM and TAM using an ultrasound unit (SonoSite, Washington, DC, USA). The ultrasound probe was dressed in a sterile cover and the injection site was disinfected. The in-plane technique was used to perform the TAPb. Thus, the needle tip was placed in the TAP and dry aspiration provided, then a test injection of several milliliters of 0.9% saline solution was injected to visualize and ensure the correct needle position. Twenty milliliters of 0.75% ropivacaine (B. Braun, Melsungen, Germany) were injected under ultrasound visualization.

## 5. Surgical Technique

The PAOs were performed by a high-volume surgeon using our specified technique. Our standard PAO approach is a less invasive, rectus tendon sparing approach with osteotomy of the anterior superior iliac spine. The skin incision follows the inguinal fold and is at least ten centimeters long. It begins in the anterior third of the iliac crest and is directed medially and distally over the ASIS in the direction of the pubic symphysis. The exposure to perform the supra- and retro-acetabular osteotomy is achieved by detaching the aponeurosis of the abdominal wall and the inguinal ligament from the iliac crest. During wound closure, we reattach the sartorius muscle and the abdominal wall to the anterior superior iliac spine and the iliac crest, respectively [20].

## 6. Outcome Assessment

Intraoperative opioid consumption, MAP, and heart rate were measured and documented at 5 min intervals. The use of other administered drugs (cafedrine/theodrenaline) was likewise documented.

## 7. Statistical Analysis

For the demographic data, mean values and ranges were calculated. Categorical variables were described with percentages. The Kolmogorov–Smirnov test was used to test for normal distribution. For normally distributed data, the t-test was used. We used the Mann–Whitney U Test for nonparametric data. Statistical significance was defined as *p* < 0.05. All statistical analyses were performed using SPSS 26 (IBM Corp., Armonk, NY, USA).

## 8. Results

The main finding of the present study was the significantly lower mean intraoperative opioid consumption in the TAPb group compared to the control group. The mean intraoperative opioid consumption in the TAPb group was 930 MED per kg bodyweight (379 to 2577), while it was 1186 MED per kg bodyweight in the control group (426 to 3214) (*p* = 0.016).

The secondary outcome parameters (MAP, heart rate, and theodrenaline/cafedrine consumption) did not significantly differ, as shown in Table 1. Eleven patients in each group (52.4% of the study group and 55% of the control group) received intraoperative theodrenaline/cafedrine.

The mean surgery time was 78.2 minutes (50 to 142) and 78.7 minutes (55 to 137) in the TAPb and control groups, respectively (*p* = 0.92). The mean LOHS was 8.7 days (7 to 15) in the TAPb group and 8.8 days (7 to 11) in the control group (*p* = 0.83). No perioperative TAPb- or surgery-related complications were observed in either group.

## 9. Discussion

This is the first study to investigate the utilization of TAPb for intraoperative pain management in PAO patients. The main finding was that intraoperative opioid consumption was significantly lower in the TAPb group compared to the control group. No perioperative TAPb-related complication was observed in TAPb group. This result is consistent with the current literature, indicating overall low complication rates for ultrasound-guided TAPb [5,6,21].

A TAPb reliably anaesthetizes the PAO surgical field, including the incision site, iliac crest, and groin [6]. Improved postoperative pain management and lower opioid consumption have been previously shown [4,22]. This fact, and the findings of this study, support our perception that surgeries of the hip and of the lower abdominal region are among the best indications for TAPb [21]. Performing a unilateral TAPb allows for the administration of higher doses of LA, increasing the analgesic potency [3].

LIA and EA are among the alternative locoregional pain management strategies for PAO patients. There are only a few studies investigating LIA use for pain management in hip joint preservation surgery. LIA is administered into the perifocal tissue during wound closure and provides analgesic effect in the immediate postoperative period; it is not used for intraoperative analgesia. EA provides reliable pain relief for the lower limbs, both intra- and postoperatively [18,23]. EA is a safe procedure and the reported complication rates are low [24]. EA is routinely used in PAO patients, however no randomized studies have been reported with regard to the efficacy of this practice [16]. EA can cause relevant motor weakness in up to 15% of cases, compromising postoperative neurological examination and mobilization [25].

Bech et al., investigated the use of LIA in a series of 53 PAO patients who received an additional surgical site catheter for prolonged LA application. No reduction in perioperative opioid consumption or improvement for pain scores was shown [26]. A further study showed lower pain scores twelve hours postoperatively following surgical hip dislocation in 20 patients given LIA (containing ropivacaine, morphine, and methylprednisolone) compared to patients administered EA and PCA [25].

Opioids have numerous side effects. Limiting their use reduces the frequency of both PONV and postoperative drowsiness, and is therefore beneficial.

We observed a relatively large standard deviation for overall intraoperative opioid consumption in both groups. This fact may be due to distinct interindividual differences for pain sensitivity and tolerance to analgesia. In contrast to existing literature investigating TAPb use in abdominal surgery, we did not observe a significant influence on circulation parameters. This may be explained by age and comorbidity differences in the study populations.

A limitation of this study can be seen in the potential bias of the study design for the LOHS. As the lower limit for LOHS after PAO in Germany is five days, an earlier discharge leads to reimbursement penalties for the surgical department. An earlier discharge after PAO, although medically possible, is not among the priorities for the clinical management of those patients. We do not provide long-term clinical follow-up, and therefore cannot assess potential mid- or long-term effects of TAPb. This can be seen as a further limitation of this study. Upcoming studies may investigate the potential effects of adding adrenaline to the LA, or the use of a TAP catheter to extend the effect of the block.

## 10. Conclusions

In conclusion, we were able to show that an ultrasound-guided TAPb is an outstanding tool to optimize pain management in PAO patients by significantly reducing the intraoperative opioid consumption.

## Figures and Tables

**Table 1:** Intraoperative circulation parameters.

Parameter	TAP Block Group	Control Group	*p* Value
MAP (mmHg)	70.3 (61 to 73)	70.3 (61 to 77)	0.135
Heart rate (per min)	60.7 (48 to 73)	59.5 (41 to 77)	0.12
Theodrenaline/Cafedrine use (mg)	52 (0 to 280)	58.5 (0 to 240)	0.62

## Data Availability

The data presented in this study are available on request from the corresponding author.

## Supplementary Materials

The following supporting information can be downloaded at: https://www.mdpi.com/article/10.3390/jcm11174961/s1, File S1: CONSORT 2010 checklist of information to include when reporting a randomised trial.
